# Combinatorial Ranking of Gene Sets to Predict Disease Relapse: The Retinoic Acid Pathway in Early Prostate Cancer

**DOI:** 10.3389/fonc.2017.00030

**Published:** 2017-03-15

**Authors:** Hieu T. Nim, Milena B. Furtado, Mirana Ramialison, Sarah E. Boyd

**Affiliations:** ^1^Faculty of Information Technology, Monash University, Melbourne, VIC, Australia; ^2^Australian Regenerative Medicine Institute, Monash University, Melbourne, VIC, Australia; ^3^The Jackson Laboratory, Bar Harbor, ME, USA; ^4^EMBL – Australia Collaborating Group, Systems Biology Institute Australia, Monash University, Melbourne, VIC, Australia

**Keywords:** prostate cancer, retinoic acid, prognosis, systems biology, The Cancer Genome Atlas, data mining

## Abstract

**Background:**

Quantitative high-throughput data deposited in consortia such as International Cancer Genome Consortium (ICGC) and The Cancer Genome Atlas (TCGA) present opportunities and challenges for computational analyses.

**Methods:**

We present a computational strategy to systematically rank and investigate a large number (2^10^–2^20^) of clinically testable gene sets, using combinatorial gene subset generation and disease-free survival (DFS) analyses. This approach integrates protein–protein interaction networks, gene expression, DNA methylation, and copy number data, in association with DFS profiles from patient clinical records.

**Results:**

As a case study, we applied this pipeline to systematically analyze the role of *ALDH1A2* in prostate cancer (PCa). We have previously found this gene to have multiple roles in disease and homeostasis, and here we investigate the role of the associated *ALDH1A2* gene/protein networks in PCa, using our methodology in combination with PCa patient clinical profiles from ICGC and TCGA databases. Relationships between gene signatures and relapse were analyzed using Kaplan–Meier (KM) log-rank analysis and multivariable Cox regression. Relative expression versus pooled mean from diploid population was used for *z*-statistics calculation. Gene/protein interaction network analyses generated 11 core genes associated with *ALDH1A2*; combinatorial ranking of the power set of these core genes identified two gene sets (out of 2^11^ − 1 = 2,047 combinations) with significant correlation with disease relapse (KM log rank *p* < 0.05). For the more significant of these two sets, referred to as the optimal gene set (OGS), patients have median survival 62.7 months with OGS alterations compared to >150 months without OGS alterations (*p* = 0.0248, hazard ratio = 2.213, 95% confidence interval = 1.1–4.098). Two genes comprising OGS (*CYP26A1* and *RDH10*) are strongly associated with *ALDH1A2* in the retinoic acid (RA) pathways, suggesting a major role of RA signaling in early PCa progression. Our pipeline complements human expertise in the search for prognostic biomarkers in large-scale datasets.

## Introduction

Large volumes of cancer genomic data are being continuously generated *via* consortia such as The Cancer Genome Atlas (TCGA) (1) and the International Cancer Genome Consortium (ICGC) (2), and optimal use of this data promises improvement to patient care (3). In particular, better characterization of the smaller subgroup of patients with poor disease outcomes will help to develop risk-adjusted treatments and potential novel therapies (4), which should significantly improve treatment selection and outcomes for patients overall.

Many large-sized gene panels have been generated to classify cancer patients into subgroups, but frequently those gene sets have poor prognostic value (5). The lack of effective biomarkers, and the failure to appropriately stratify patients according to disease severity and prognosis, leads to an increased burden on both the patient and the health-care system, with inappropriate, under- and over-treatment of patients (6). With an ever-increasing number of prognostic gene signature reports (~250 yearly, based on a PubMed search with query [((“gene signature” OR “gene signatures”) AND “cancer”)]), the oncology research community would benefit from a systematic evaluation method to benchmark these diverse studies.

Recent studies of different cancer patient cohorts have incorporated some machine learning techniques such as decision trees (7) and Bayesian belief networks (8, 9). These techniques are computationally intensive, frequently rely on heuristics to explore the gene-set space, and commonly suffer from small-sized patient cohorts (10).

In our experience working with clinical oncologists/pathologists, an important result of the computational method is to conclusively demonstrate the optimality of the discovered gene set based on standard clinical measures in an exhaustive search. As an example of non-exhaustive search, a recent high-impact study by Irshad et al. in newly diagnosed prostate cancer (PCa) examines only 3-gene combinations in a 19-gene set, i.e., 969 out of 524,287 possibilities (7). In our proposed pipeline, we use gene/protein (from here onward referred to simply as gene) interaction network to generate a core gene set, then combinatorially generate and rank gene sets based on the standard Kaplan–Meier (KM) log-rank *p*-value, and finally examine the clinical relevance of the optimal gene set (OGS) using ANOVA of Cox proportional hazard models. Valuable features of our pipeline are its deterministic, unbiased, and clinician-intuitive nature.

In PCa, the biology is complicated by a high degree of both intra-patient (11) and inter-patient heterogeneity (12), and progress in treatment has been hampered by a lack of predictive biomarkers (13). The current prognostic protocols, which combine Gleason score, prostate-specific antigen (PSA), and clinical stage, have limited value in predicting outcome (14, 15). There is a pressing need for validated biomarkers that provide objective assessment of the prostate tumor biology and prognostic stratification, especially in early PCa (14).

As a case study, we applied our pipeline to a potential biomarker candidate for early PCa, the retinoic acid (RA) *ALDH1A2*, which we have previously identified and experimentally validated as being worthy of further biological characterization (16). Vitamin A (retinol) is a lipid-soluble organic compound that plays essential roles in embryonic development, cell proliferation, differentiation, and apoptosis (17). It is normally obtained either directly through diet or indirectly through the conversion of β-carotene in the body through oxidation. Within the cell, vitamin A undergoes multistep metabolic processing, to produce RAs such as *ALDH1A2*. The RA then binds to its nuclear hormone receptors, forming active heterodimers that modulate expression of downstream RA target genes by binding to DNA regions named RA response elements.

The biology of *ALDH1A2* is complex, and its roles in cancer are being increasingly explored (18–20). Even without the exact mechanisms being fully understood yet, we are able to use the putative role of *ALDH1A2* in cancer to derive a core gene set from *ALDH1A2*-interacting partners, using available literature and curated databases. With our novel data-mining algorithm, we systematically evaluate combinatorial subsets of this gene signature, in relationship to disease-free survival (DFS) and other relevant clinical parameters including the subjective histological grading called Gleason score. We arrive at an optimal gene signature that, when aberrantly expressed, is strongly associated with PCa relapse.

## Methods

### Data Used in This Study

This study was exempt from ethical review by Monash University Human Research Ethics Committee (MUHREC) as the research involved only de-identifiable data about human beings. De-identified PCa patient data were retrieved and processed from TCGA database, specifically the “TCGA Prostate Adenocarcinoma” study, accessed using the application programming interface from cBio Cancer Genomics Portal (21). Genomics data were downloaded the data from the European Genome-phenome Archive (EGA) through approved access, with accession number EGAD00001001329 (22). Gene expression data were obtained *via* the NCBI Gene Expression Omnibus (GEO) database with accession number GSE35988 (23). Table 1 summarizes the data sources gathered and integrated in this study. All patient data were uniformly assessed in subsequent bioinformatics and biostatistical analyses.

**Table 1:** **Data used in this study**.

Database	Dataset	PMID	Platform
BIOGRID 3.4	BIOGRID-ALL-3.4.138	25428363	Two-hybrid, affinity capture MS, and genetics
STRING 10	protein.link.detailed.v10	25352553	Protein–protein interaction network and text mining
TCGA	PRAD	26544944	RNA-Seq, DNA copy number, and clinical profile
EGA/ICGC	EGAS00001000682	25066126	DNA methylation
Ingenuity^®^ Pathway Analysis	Ingenuity Knowledge Base	24336805	Causal network and interaction network
NCBI GEO	GSE35988	22722839	Gene expression
DAVID 6.7	DAVID Knowledgebase	19131956	Gene ontology annotation

### Bioinformatics Analysis

RNA-sequencing data were normalized as previously described (21), with *z*-statistics calculated based on relative expression levels versus population mean: |*z*| > 1.96 (i.e., outside 95% confidence interval) indicates altered expression. Microarray analysis of Agilent platform data was performed as described previously (24). Genome-wide DNA methylation profiling from the Illumina 450K platform data was performed following the RnBeads processing pipeline (25). Subset-quantile normalization was performed using SWAN (26). Probes with missing samples or detection *p*-value below 0.01 or containing single nucleotide polymorphism were excluded. Beta values were used to represent methylation levels.

### Clinical Association with DFS Analyses

Gleason score was used as a categorical variable. Other clinical covariates included counts of examined lymph nodes, most recent PSA score, and patient age. Outcomes were analyzed by KM analysis with log-rank test. Univariate analysis and multivariate analysis for determining prognostic association between gene signatures and clinical parameters were performed using Cox proportional hazards regression. KM and Cox regression analyses were performed using R version 3.2.3 *via* the “survival” package (27).

### Gene Set Generation and Ranking Based on Clinical Profiles

**Figure 1:**
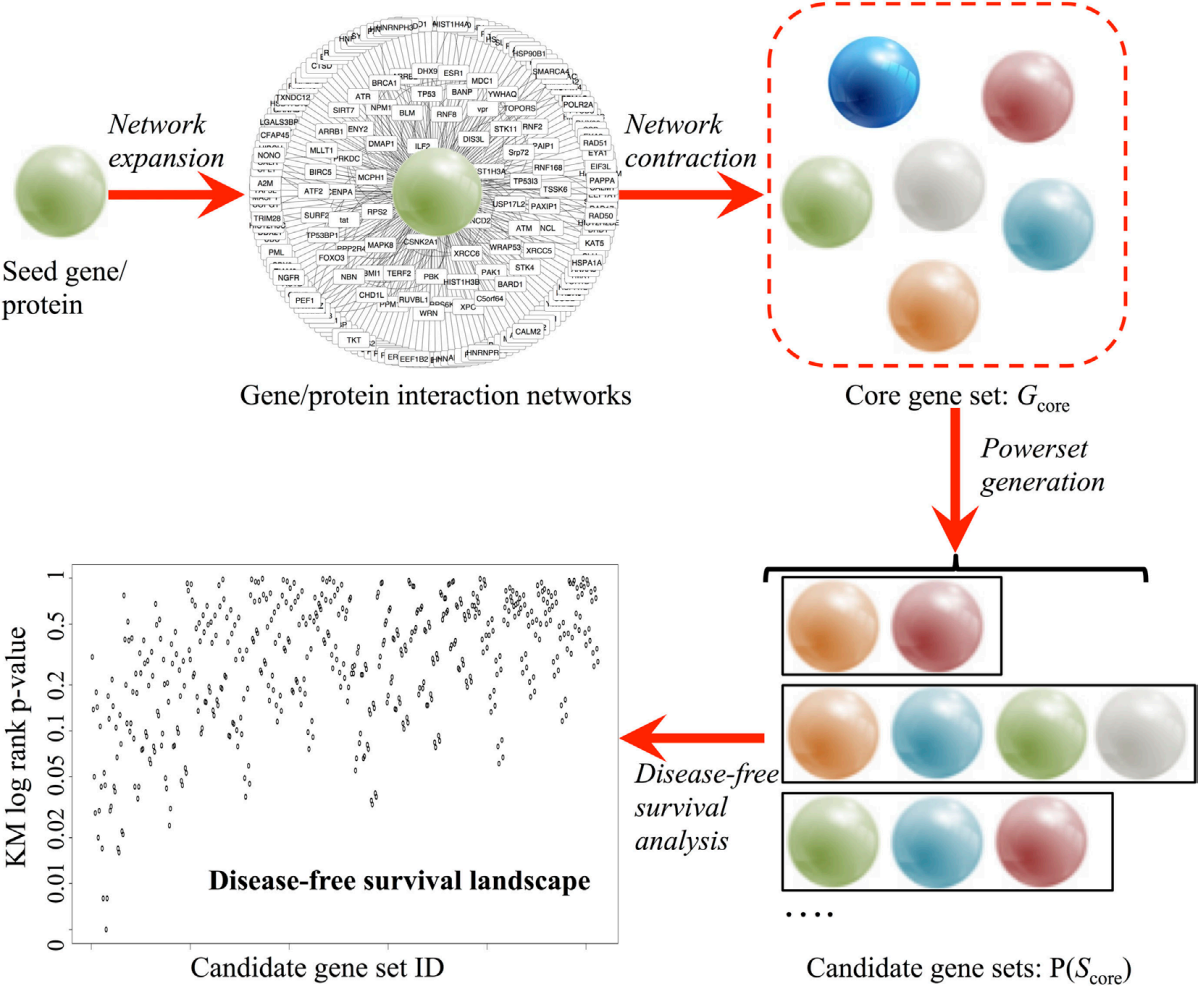
**Pipeline of the combinatorial ranking procedures, developed to systematically explore and evaluate gene sets based clinical relevance**. A core gene set (*G*_core_) is derived in a two-phase procedure: (1) network expansion using Ingenuity^®^ Pathway Analysis and (2) network contraction by verifying the individual network links in BIOGRID 3.4 and STRING 10 databases. Power set generation populates all combinatorial gene sets based on *G*_core_. Finally, disease-free survival analysis ranks all candidate gene sets based on prognostic values.

A comprehensive survey of all the available data from established public data repositories and published literature and abstracts was used to produce an unbiased assessment of the genes involved in the seed gene of interest (Figure 1).

In the network expansion phase, a preliminary interaction network was generated from the Ingenuity^®^ Pathway Analysis [Ingenuity Pathway Analysis (IPA)—Qiagen] database using the seed gene as query. Using the “Export” option, a text (.TXT) file containing all interacting partners of the seed gene was obtained. Using Microsoft^®^ Excel™ 2013 software, three columns were extracted: the official gene symbol (e.g., *Tp53*), gene description (e.g., tumor protein p53), and synonyms (e.g., *Bbl, Bcc7, Bfy, Bhy, Brp53*, and *Brp53*). The synonyms are then used for removing duplicate results in IPA output *via* R script.

In the network contraction phase, the preliminary network was first filtered through the BIOGRID 3.4 database (Table 1) (28). BIOGRID database snapshot in tab-separated text format was downloaded from https://thebiogrid.org (version 3.4.138, 348 MB). Using Microsoft^®^ Excel™ 2013 software, three columns were extracted: interactor A (e.g., *Tp53*), interactor B (e.g., *Mdm2*), and interaction types (e.g., affinity capture-luminescence, two-hybrid, etc.). Custom R script extracted all data rows containing the seed gene and interacting partner found earlier from IPA. Next, the top interacting partners were extracted from multiple lines of evidence from the literature using the STRING v10 database (Table 1) (29). STRING v10 data access was requested for academic use (http://string-db.org). Upon approval, database snapshot in tab-separated text format was downloaded (protein.links.full.v10.txt.gz, 17.8 GB). A custom R script was used to extract all data rows containing the seed gene and interacting partners found earlier from IPA. Two sets of matching results from BIOGRID and STRING were combined, and the relationship between seed gene and interacting partners was then labeled as co-expression, text mining, database interrogation, and experimental data. The interacting partners of the seed gene with only one evidence were filtered out to generate a stringent list of interacting partners (*G*_core_). A functional analysis of the genes in *G*_core_ was conducted using DAVID v6.7 (30) based on pathway and gene ontology annotations [KEGG pathway (31), biological process, cellular component, and molecular function] to confirm the biological pathway relevance of *G*_core_.

Upon obtaining a curated set of genes, power set (i.e., set of all subsets) generation is performed using the R *powerset* package. For each set of gene, a validation pipeline was executed based on PCa patient data (TCGA, ICGC, and GEO) using the bioinformatics analysis protocol described earlier (R code described in Data S1 in Supplementary Material and available on GitHub). In brief, genomic data from tumor and healthy tissue were downloaded. Patients were grouped according to altered expression (|*z*| > 1.96) status, as defined earlier. For TCGA data, clinical parameters were also downloaded for DFS analysis. Based on the calculated KM *p*-values, the algorithm ranks all candidate genes based on prognostic probability in the TCGA early PCa patient cohort.

## Results

*ALDH1A2* is a key player in the RA pathway and retinoid metabolism, both known to be important in homeostasis and cellular function (32, 33), the disruption of which leads to various health problems including PCa (34, 35). In our case study, we start with *ALDH1A2* to generate our core gene set.

### Generation of a Core Gene Set *via* Data Integration

**Figure 2:**
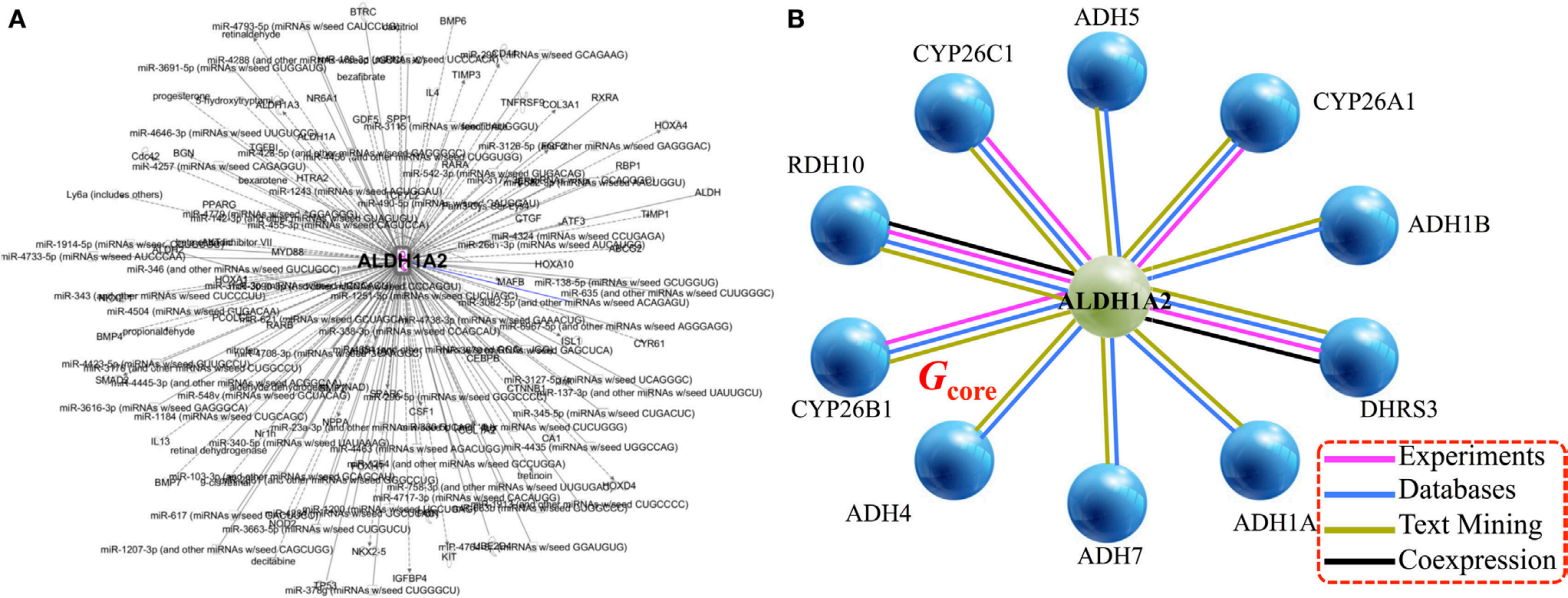
**Gene/protein interaction network of *ALDH1A2***. **(A)** Network expansion phase: Ingenuity Pathway Analysis tool gives 279 interaction partners of *ALDH1A2*. **(B)** Network contraction phase: BIOGRID 3.4 and STRING 10 databases reduce the 279-node *ALDH1A2* network down to 11 nodes (genes/proteins), with a minimum of two lines of evidence (indicated with colored lines).

Applying the pipeline using *ALDH1A2* as the seed (Figure 1), we obtained a large gene interaction network (Figure 2A), which was then refined to a core gene set (*G*_core_) of 11 genes (Figure 2B). DAVID gene ontology analysis shows that all 11 genes are involved in both “retinol metabolism” (KEGG pathway) and “oxidation reduction” (GO biological process). We analyzed the expression and methylation levels of *G*_core_ independently in two landmark PCa datasets: Grasso et al. (23) (Table 2) and Brocks et al. (22) (Table 3). The individual genes in *G*_core_ were strongly associated with differential expression (Table 2) but not differential methylation (Table 3) between tumor and normal patients. However, it is possible that combinations of these individual genes may have prognostic value, so these were also further assessed, as described in the following.

**Table 2:** **Differential gene expression analysis between cancer and normal samples based on Grasso et al. dataset (23)**.

Gene symbol	Probe ID	Adjusted *p*-value	Log fold-change
*ALDH1A2*	A_24_P73577	2.24E−15	−3.811791
*CYP26A1*	A_23_P138655	3.35E−03	2.7341369
CYP26B1	A_23_P210100	6.86E−02	−1.3187345
*RDH10*	A_32_P25050	1.57E−01	−0.7989623
ADH5	A_24_P260346	2.68E−13	−2.1221184
DHRS3	A_23_P33759	1.16E−01	−0.4794662
ADH4	A_23_P30098	3.91E−01	0.592659
ADH1B	A_24_P940469	4.15E−03	2.0103106
ADH1A	A_24_P291658	1.58E−02	1.669169

**Table 3:** **Differential methylation analysis between tumor and normal samples based on Brocks et al. dataset (22)**.

Gene symbol	ENSEMBL ID	Adjusted *p*-value	Log fold-change
*ALDH1A2*	ENSG00000128918	0.800930711	−0.033209504
CYP26C1	ENSG00000187553	0.800930711	−0.074378124
*CYP26A1*	ENSG00000095596	0.800930711	−0.035321392
CYP26B1	ENSG00000003137	0.800930711	−0.048280355
*RDH10*	ENSG00000121039	0.800930711	0.015297498
ADH5	ENSG00000197894	0.800930711	0.02746407
DHRS3	ENSG00000162496	0.800930711	0.06188264
ADH7	ENSG00000196344	0.887042456	0.004002081
ADH1B	ENSG00000196616	0.800930711	0.085607147
ADH1A	ENSG00000187758	0.800930711	0.116789434

### OGS Expression Profiles Based on Predictive Power Using the TCGA Patient Cohort

The TCGA dataset contained 491 PCa cases for which there was clinical information, and of those, 92 patients (18.7%) had relapsed. A comparison between relapsed patients and patients with DFS did not identify any significant differences in the clinical characteristics of age, number of lymph nodes removed, and most recent PSA value (all *p*-values ≥0.05 using heteroscedastic unpaired *t*-test; Table 4).

**Table 4:** **Heteroscedastic unpaired *t*-test of 491 patients in The Cancer Genome Atlas cohort shows no difference between age, number of lymph nodes, and most recent prostate-specific antigen (PSA) results with reference to disease relapse**.

Clinical parameters	No relapse (*n* = 399)	Relapse (*n* = 92)	*p-*Value
Age	60.877 (6.999)	61.554 (5.944)	0.343
Number of lymph nodes	11.538 (9.129)	13.095 (11.892)	0.265
Most recent PSA results	0.822 (3.605)	1.865 (5.301)	0.085

*Means and SDs are shown*.

**Figure 3:**
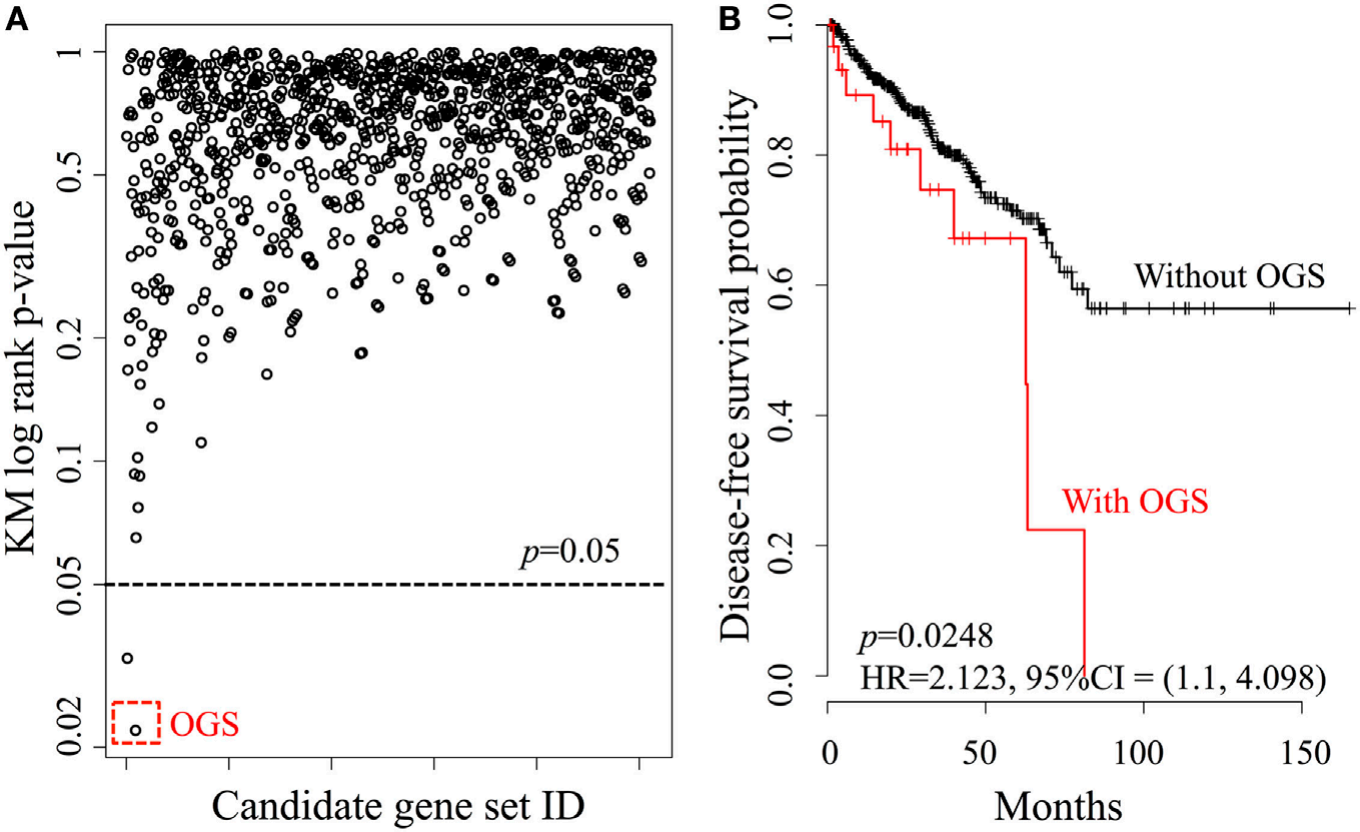
**Systematic analysis of all candidate gene sets, generated from the power set of 11 genes in *G*_core_**. **(A)** The DFS Kaplan–Meier (KM) log-rank *p*-value landscape from the *ALDH1A2*-derived candidate gene sets. The optimal gene set (OGS) according to KM log-rank *p*-values is indicated (red dashed box). **(B)** KM log-rank survival curves of *n* = 491 patients in the TCGA cohorts with respect to the presence or absence of aberrant expression (based on *z*-statistics) of genes in the OGS.

We investigated the relationship between DFS and every possible candidate gene set based on the core gene set *G*_core_, defined as the power set of *G*_core_. The KM log-rank statistic was used for unbiased exploration of these gene subsets in correlation with DFS, producing a DFS landscape (Figure 3A). Surprisingly, the KM *p*-values were found to be statistically significant for only two gene sets (Figure 3A, below dashed line), at a probability of 2/2047 = 0.1%. The OGS comprises *CYP26C1* and *RDH10*, both of which coordinate tightly with *ALDH1A2* to control RA activities (36, 37).

We then compared DFS in the patient cohort (*n* = 491) with altered expression of the two genes in the OGS (Figure 3B). Being positive for OGS, defined as having significantly altered expression of any or all of the genes in the OGS signature, was associated with statistically significant poor survival (log-rank *p*-value = 0.0248, HR = 2.213, 95% CI = 1.1–4.098). A strong correlation is seen between OGS and DFS: patients with OGS have median DFS of 62.7 months (Figure 3B, red curve) while >55% patients without OGS are still disease-free after 150 months (Figure 3B, black curve).

### Complementarity of Prognostic Value of OGS with Traditional Clinical Measures

We performed univariate Cox regression to investigate the association between OGS and Gleason score (Table 5). Gleason score, devised in the 1960s and 1970s by Donald Floyd Gleason, is one of the most well-established clinical measures of PCa disease status, where the conventional scale of 6–10 (hereby referred to as OldGleason) is routinely practiced (38). Recently, John Hopkins researchers challenged this old scale and proposed a new scale of 1–5 (hereby referred to as Gleason) that was validated to exhibit better prognostic values (15). In agreement with recent studies (7), low grade Gleason scores (OldGleason 6 and 3 + 4) are not predictive of disease relapse (Table 5). In contrast, whether a patient has altered OGS expression (based on gene expression, |*z*| > 1.96, see Bioinformatics Analysis) can predict DFS based on Cox regression analysis (Table 5, *p* = 0.0248, HR = 2.123). Further, ANOVA analysis of the multivariable Cox regression model with OGS plus Gleason score (Gleason + OGS, Table 6) shows that adding the OGS variable significantly improve the predictive power of Gleason score alone in a univariable Cox regression model (*p* = 2.19 × 10^−11^ at 4 degrees of freedom).

**Table 5:** **Univariate Cox regression analysis of Gleason score and optimal gene set (OGS), with respect to disease-free survival**.

Clinical parameters	Univariate HR (95% CI)	*p*-Value
Gleason 1 (OldGleason ≤ 6)	Reference	Reference
Gleason 2 (OldGleason = 3 + 4)	3.638 (0.473, 27.98)	0.21472
Gleason 3 (OldGleason = 4 + 3)	5.223 (0.6788, 40.19)	0.11233
Gleason 4 (OldGleason = 8)	9.173 (1.1997, 70.13)	0.03273*
Gleason 5 (OldGleason ≥ 9)	20.826 (2.8802, 150.58)	0.00263*
OGS	2.123 (1.1, 4.098)	0.0248*

*p-Value < 0.05.

**Table 6:** **ANOVA analysis for multivariable Cox regression models of Gleason score and/or optimal gene set (OGS)**.

Clinical parameters	Log (likelihood)	Chi-square	Degree of freedom	*p-*Value
Gleason	−494.69	Reference	Reference	Reference
Gleason + OGS	−466.78	55.816	4	2.191 × 10^−11^*

*p-Value < 0.05.

## Discussion

As the number of large-scale genomics datasets exponentially increases due to decreasing experimental costs, current limitations reside in our capacity to extract relevant information. Our study illustrates a novel pipeline applicable to any range of disease cohorts that can assist in mining these datasets in a robust and unbiased way to generate clinically relevant knowledge. Combinatorial enumeration of all possible subsets of *n* genes with DFS helps isolate *k* gene sets based on statistical significance (i.e., KM log-rank *p*-value < 0.05). From there, we are able to identify an OGS signature, whose dysregulation can be associated with DFS in early stage PCa patients (Figure 3). We illustrate this process with *ALDH1A2*, where by using this RA as a seed for the data-mining pipeline, we identify an initial set of *n* = 11 genes, which is reduced by statistical significance, first to *k* = 2 gene sets, and then refined to an OGS containing just two genes. This optimal gene signature has significant predictive power of relapse, both alone and in combination with the traditional histological Gleason score.

The decision to use *ALDH1A2* as a seed for our case study comes from the increasing evidence for the role of RAs in mammalian homeostasis and disease, especially the intimate association of the RA signaling pathway with a variety of cancers, including leukemia, neuroblastomas, and carcinomas, as well as gastric, ovarian, lung, breast, colon, rectal, pancreatic, and PCas (18–20). In PCa, a previous study has found hypermethylation of *ALDH1A2* in cancer cell lines subjected to treatment with the chemotherapeutic agent 5-aza-2′-deoxycytidine (18). Hypermethylation of *ALDH1A2* led to reduced gene expression in PCa cell lines. Moreover, *ALDH1A2* levels were also reduced in human primary prostate tumors when compared with normal prostate tissue. Reduced expression of *ALDH1A2* also correlated with shorter recurrence-free survival of patients, suggesting that *ALDH1A2* may in fact be a tumor suppressor gene for PCa. A second study using an adenocarcinoma prostate model confirmed reduction of *ALDH1A2* in prostate tumors in mice (33). This study was further supported by measuring *ALDH1A2* protein levels in prostate tissue from PCa patients, where PCa tissue samples showed reduced *ALDH1A2* expression compared with healthy tissue. Finally, the *ALDH1A2* case study has been chosen because of its potential significance other disease models, as we have previously demonstrated that the *ALDH1A2* pathway is involved in a completely different disease context, i.e., cardiac fibrosis (39, 40).

Our case study results are not only consistent with the evidence for the role of *ALDH1A2* in PCa but also show that the *ALDH1A2* pathway could potentially be used as biomarker for treatment selection: aberrant expression of genes involved in the regulation of *ALDH1A2* defines a patient group associated with a significantly high risk of relapse, thereby facilitating stratification of patients to ensure the appropriate individualized selection of therapy. Combining the OGS with clinical parameters, especially Gleason score, further increases discrimination between relapsing and non-relapsing patients.

The combinatorial ranking procedure can be applied to other cancers, with appropriate adjustment based on the available datasets. We performed combinatorial ranking on the TCGA Breast Cancer dataset with 10 genes of *G*_core_ (*ADH1B* was excluded due to missing data in this cohort). The metrics used was KM log-rank *p*-values for overall survival, rather than DFS. The procedure returned three significant gene sets with *p* < 0.05 (Figure S1 in Supplementary Material), where the OGS* contains *ADH5, ADH7*, and *CYP26A1*. Being positive for OGS* was associated with statistically significant poor overall survival (log-rank *p*-value = 0.0201, HR = 1.245, 95% CI = 1.035–1.497).

The major limitations of this study relate to the data. In this cohort, patients were seen at multiple different institutions, which could lead to some biases in sample collection and data collection and processing. Added to this, overall survival information is not available, which therefore limits the analysis to DFS. This is a significant issue in diseases such as PCa, where there may be a long period of time prior to relapse. Our analysis also does not take in to account the type of treatment that was administered, which could affect patient outcomes. Finally, our automated data-mining approach has a benefit of being unbiased, but at the same time we may lose some of the expertise-driven analyses that are emerging from studies of individual genes.

Despite these limitations, our *ALDH1A2*-derived OGS is nevertheless highly predictive of PCa relapse, and of particular note it is predictive in the context of early PCa, where decisions around treatment can be most difficult in terms of being appropriate and proportional to the disease severity and prognosis. The pipeline is automated, which allows a large-scale and unbiased assessment of the available data, such that just a single seed gene can be used to generate then rank very large numbers of gene panels, and is designed to be intuitive for clinicians. The case study illustrates the power of the pipeline with PCa but can the technology be applied to any cancer, or indeed any other disease, especially where clinical data are available to assess the prognostic value of the gene panel, ahead of clinical assessment, and validation of the derived optimal gene signature.

## Author Contributions

HTN designed the study, acquired, analyzed, and interpreted the data. MF, MR, and SEB designed the study and interpreted the data with expertise in genetics, bioinformatics, and systems biology, respectively. All authors contribute to writing the manuscript. HTN, MF, MR, and SEB all met the four criteria for authorship as listed below: substantial contributions to the conception or design of the work; or the acquisition, analysis, or interpretation of data for the work; and drafting the work or revising it critically for important intellectual content; and final approval of the version to be published; and agreement to be accountable for all aspects of the work in ensuring that questions related to the accuracy or integrity of any part of the work are appropriately investigated and resolved.

## Conflict of Interest Statement

The authors declare that the research was conducted in the absence of any commercial or financial relationships that could be construed as a potential conflict of interest.

## Supplementary Material

Figure S1 **Systematic analysis of all candidate gene sets in The Cancer Genome Atlas (TCGA) breast cancer cohort (*n* = 2,509), generated from the power set of *G*_core_**. Out of 11 genes in *G*_core_, the *ADH1B* gene was excluded due to missing data from this cohort, leaving 10 remaining genes for power set generation. **(A)** The overall survival KM log-rank *p*-value landscape from the *ALDH1A2*-derived candidate gene sets. The optimal gene set (OGS*) according to KM log-rank *p*-values is indicated (red dashed box), which contains three genes: *ADH5, ADH7*, and *CYP26A1*. **(B)** KM log rank overall survival curves of patients in the TCGA breast cancer cohorts with respect to the presence or absence of aberrant expression (based on *z*-statistics) of 10 genes in OGS*.

Data S1 **R script for executing the combinatorial ranking pipeline is maintained *via* GitHub at https://github.com/nimt0001/CombinatorialRank**. The code performs combinatorial ranking based on a specific patient cohort from TCGA. As the TCGA database is updated regularly, results from the provided R script can change from run to run and should be used only as a reference.
