# Supplementary figures and images for: Combinatorial Ranking of Gene Sets to Predict Disease Relapse: The Retinoic Acid Pathway in Early Prostate Cancer
Source: Front Oncol. 2017 Mar 15;7:30. doi: 10.3389/fonc.2017.00030 (PMC5350134; doi:10.3389/fonc.2017.00030)

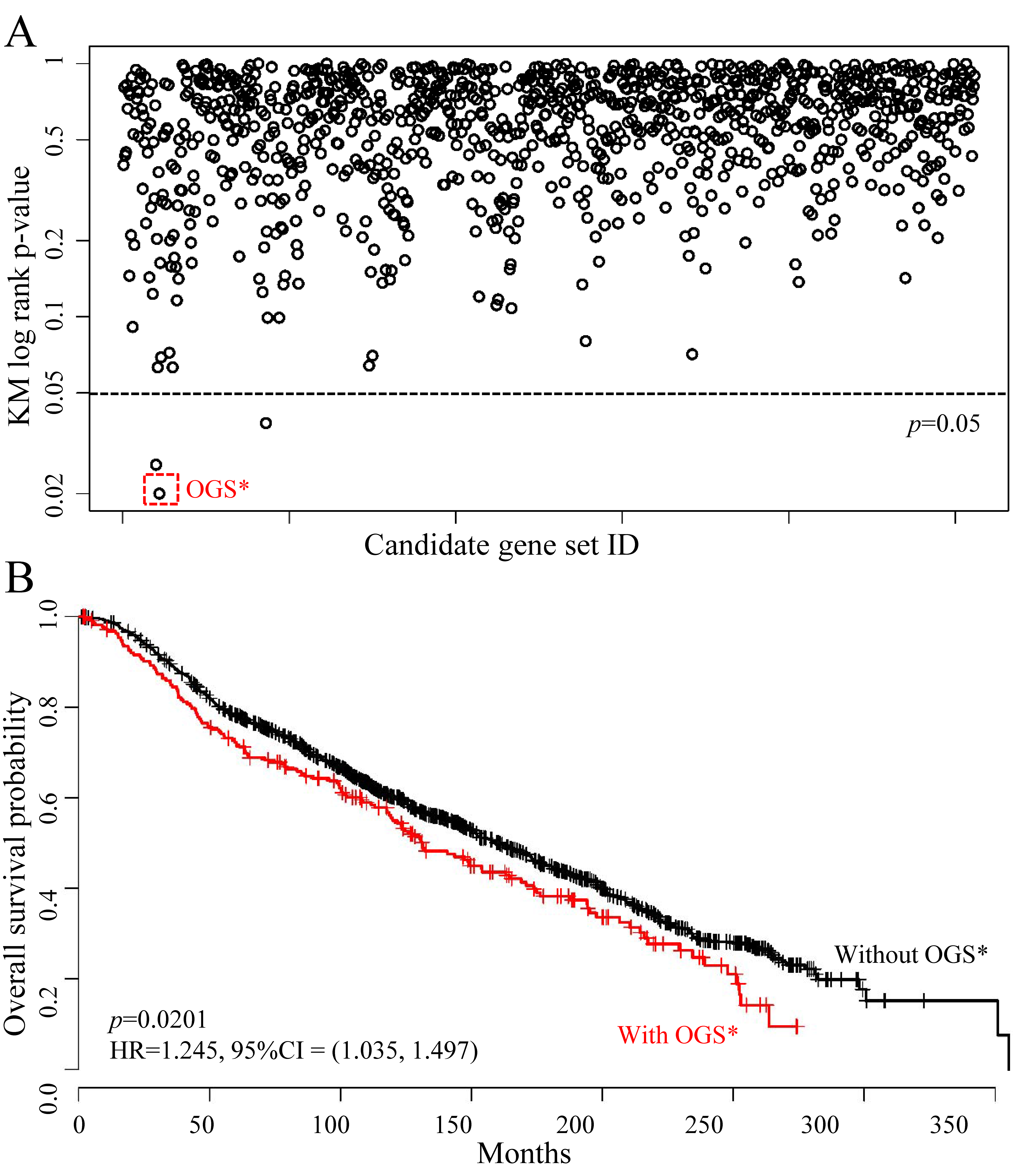

Supplement: Figure S1 — Systematic analysis of all candidate gene sets in The Cancer Genome Atlas (TCGA) breast cancer cohort (n = 2,509), generated from the power set of Gcore. Out of 11 genes in Gcore, the ADH1B gene was excluded due to missing data from this cohort, leaving 10 remaining genes for power set generation. (A) The overall survival KM log-rank p-value landscape from the ALDH1A2-derived candidate gene sets. The optimal gene set (OGS*) according to KM log-rank p-values is indicated (red dashed box), which contains three genes: ADH5, ADH7, and CYP26A1. (B) KM log rank overall survival curves of patients in the TCGA breast cancer cohorts with respect to the presence or absence of aberrant expression (based on z-statistics) of 10 genes in OGS*. [file image_1.tiff]
